# BabblePlay: An app for infants, controlled by infants, to improve early language outcomes

**DOI:** 10.1016/j.apacoust.2019.107183

**Published:** 2020-05

**Authors:** Helena Daffern, Tamar Keren-Portnoy, Rory A. DePaolis, Kenneth I. Brown

**Affiliations:** a AudioLab, Department of Electronic Engineering, University of York, United Kingdom; b Department of Language and Linguistic Science, University of York, United Kingdom; c Communication Sciences and Disorders, James Madison University, United States; d Department of Music, University of York, United Kingdom

**Keywords:** App development, Acoustic analysis, Real time feedback, Babble

## Abstract

This project set out to develop an app for infants under one year of age that responds in real time to language-like infant utterances with attractive images on an iPad screen. Language-like vocalisations were defined as voiced utterances which were not high pitched squeals, nor shouts. The app, BabblePlay, was intended for use in psycholinguistic research to investigate the possible causal relationship between early canonical babble and early onset of word production. It is also designed for a clinical setting, (1) to illustrate the importance of feedback as a way to encourage infant vocalisations, and (2) to provide consonant production practice for infant populations that do not vocalise enough or who vocalise in an atypical way, specifically, autistic infants (once they have begun to produce consonants). This paper describes the development and testing of BabblePlay, which responds to an infant’s vocalisations with colourful moving shapes on the screen that are analogous to some features of the infant’s vocalization including loudness and duration. Validation testing showed high correlation between the app and two human judges in identifying vocalisations in 200 min of BabblePlay recordings, and a feasibility study conducted with 60 infants indicates that they can learn the contingency between their vocalisations and the appearance of shapes on the screen in one five minute BabblePlay session. BabblePlay meets the specification of being a simple and easy-to-use app. It has been shown to be a promising tool for research on infant language development that could lead to its use in home and professional environments to demonstrate the importance of immediate reward for vocal utterances to increase vocalisations in infants.

## 1 Introduction

Past research (e.g., [24]; [32], [22], [35] has shown that babbling supports infants’ first word production: Infants’ early canonical babble (production of language-like consonant-vowel [CV] syllables) affects the way they listen to speech [7] and the types of consonants and syllable structures most frequent in vocalisations at the babbling stage are those used most often in children’s earliest words [32], [35]. It is also accepted that infants who start to produce consonants reliably in babble earlier also produce words earlier [22], [21].

The aim of this project was to supply infants with visual reinforcement, via a tablet device, as a way of encouraging more vocal exploration. Our assumption was that, once a child has begun to produce canonical babble, or CV syllables, then an increase in volubility will bring with it, among other kinds of vocalisations, an increase in canonical babble. This increase, or intensified practice in consonant production, should lead to more rapid consonant production mastery. Previous research, as mentioned above, suggests that earlier consonant mastery would lead to earlier language learning. It is known that it is possible to increase a motorically available rhythmic behavior in infants using visual reinforcement: Rovee-Collier and colleagues have shown that even 2-month olds will increase spontaneous kicking in order to activate a mobile [27].

Previous attempts have been made to create tools to encourage or improve infant vocalisations, often targeting populations with delayed language development. A now expired patent describes a hardware-based system designed to immediately replay an infant’s vocal utterances back to the infant over speakers alongside an interesting visual reinforcement displayed on a computer screen [3], [4].

A more recent software-based system, developed as part of the Spoken Impact Project (SIP) [11] has been specifically developed to improve language outcomes for children with Autism Spectrum Disorder (ASD), and has tested the use of visual and auditory responses with a small delay or at the end of an utterance to encourage vocalisations [14]. Specifically aimed at facilitating multisyllabic speech production, VocSyl was developed to allow a clinician to provide a visual ‘template’ image (produced with their own vocalisation) that could then be overlayed with the visual response from the child’s vocalisation [13]. VocSyl was designed and tested with children diagnosed with ASD or with speech delays as well as typically developing children alongside their carers with encouraging results [12].

Another software-based system targeting young infants with developmental speech delays, VisiBabble, visually rewards an infant for target syllables. VisiBabble used an acoustic analysis approach to identify syllable-like utterances using five of the acoustic landmarks identified by Stevens in phoneme recognition [29]. User testing of the VisiBabble system was reported on a small sample of 5 children with severe expressive impairments aged 2.5 to 7.5 years old [10]. For all of the children there was a rise in the number of utterances and the variety of syllables produced during interactions with VisiBabble relative to the periods when the screen was black and unresponsive. This suggests that the visual rewards provided by VisiBabble led to an advance in vocalising. In a longer-term study, a sample of 3 infants of varying age and with different developmental impairments used VisiBabble for eight minutes a day over an average of 17 days in a 10 week period and showed an increase in the frequency, complexity and/or variety of their vocalisations after this intervention period, and generalised the new vocal behaviour to other, non-intervention, situations [9]. However, whilst there is mention that ‘the system rarely responded to noise or whispering’ [8], it isn’t clear from the available literature on VisiBabble how the system itself was tested, in terms of its accuracy in identifying syllabic utterances made by the infant. Whilst there were several iterations of this system it seems that it has not been developed since these initial publications.

The systems discussed above are all designed as desktop computer activities and have mainly been targeted at older children with delayed speech and language development. With the rapidly growing popularity of mobile devices and in particular electronic tablets, there is a new opportunity to revisit the possibilities of producing an accessible, easy to use tool that encourages vocalizations in infants. There is an acknowledged increase in the engagement of younger infants (under 1 year) with these technologies (e.g., 52% of 6–11 month-olds in the UK are reported to use touchscreens [N = 134]: [1], as are 92% of all lower-SES infants in a US sample [N = 51]: [16]).

However, current games and apps do not treat infants as active agents who influence their own surroundings, but as passive recipients of external stimuli: Interactive apps designed for infants require the infant to respond to some stimulus (e.g. touch a picture) and no app currently exists, as far as we know, which requires or allows the infant to initiate the interaction. Producing an app which responds to the vocalisation of the infant empowers them to be in control of the game and learn the contingency between the behavior and the visual response.

Infants at risk for ASD and children diagnosed with ASD have atypical babble as regards rhythm [25] and consonant use [26]. Between ages 6 and 12 months, infants at risk for autism produce fewer speech-like and more non-speech-like (e.g., squeals, laughs, cries, growls) vocalisations than infants at low risk [28], [26]. They also receive fewer contingent (within 1 s) parental responses to their vocalisations than typically developing peers [34], presumably because their vocalisations are less speech-like. Encouraging more typical-sounding (speech-like) vocalisations in such infants through a visual screen display that does not rely on social interaction or adult facial expression as a positive reinforcer may be beneficial for these infants (who engage less in eye contact in their first year than typically developing infants: Clifford & Dissanayake [5], but see [33]. Such a change in vocalisation type may in turn lead to more contingent parental responses.

The approach taken in this project is quite different from that taken by the creators of LENA (Language Environment Analysis system, [20], a system which is widely used to study infant vocalisations. The LENA system allows researchers to record the sounds an infant hears and produces over many hours without the need for an external observer. It also estimates the amount of vocalisations produced by the infant and the adults around it. However, LENA analysis takes place off-line, once the recordings have been downloaded. They can be used to provide *parents or researchers* with a picture of the language environment of the infant. They do not include a visual interface which can provide *the infants* with feedback on their own vocalisations on-line. As such, BabblePlay and LENA complement each other, in terms of the information they provide, when they provide it and who can make use of it.

In this project we set out to develop an iPad app that would encourage more intensive babbling in infants, by supplying them with attractive visual feedback, using a tool which is non-invasive and requires no engagement on behalf of the parents (beyond switching it on and off). The purposes of the app are twofold: firstly it is intended as a tool for research, whereby the causal relationship between increased consonant production and early language acquisition can be investigated. Secondly, the app is designed to be used directly by families with their infants as a means to promote vocal development. In particular, it can be used to demonstrate to families the importance for infants of receiving feedback on their early vocalisations.

### 1.1 Contributions

This paper provides the following contributions:

1) We introduce a new app, BabblePlay, specifically developed to encourage vocal utterances. The app is designed so that infants under a year old can control it, by using their voice to initiate the appearance of moving images on the screen.
2) We demonstrate that the app is in good agreement with humans in detecting an infant’s vocalisations as distinct from bangs and other non-harmonic environmental sounds, high pitched squeals and unvoiced utterances.

We show, using data collected from a feasibility study reported elsewhere, that infants can learn the contingency between their voiced utterance and the app’s response.

## 2 Specification

This section first presents the background literature which informed the design of the interface to be suitable for the target user and the system aims, followed by a breakdown of the resulting technical specifications of BabblePlay.

### 2.1 Background: context for interface design

The target audience of this project in the first instance is typically developing infants between 5 and 10 months old. Future developments will target infant populations at risk of language delay for whom more practice with speech-like vocalisations would be beneficial, e.g., infants at risk for ASD.

Canonical babbling typically develops between 6 and 8 months [31]) with an average age of about 6 months [24]. Therefore, a key requirement of the app’s design was for the visual display to be attractive to typically developing infants of this age, so that they would be incentivised to remain engaged with the screen rather than their wider surroundings. By age 6 months, infants are capable of following a moving object with their gaze and make predictive eye movements, they prefer moving objects to stationary ones, and they can distinguish between types of object movement (side-to-side vs. rotating). Infants of this age also prefer to look at objects that are patterned (rather than with a solid surface), and can already perceive colours as belonging to categories of hue [15], [2].

Studies on contingency learning in infants showed that long latencies between the infant’s action (usually limb movement) and a resultant visual display led to less effective learning: Millar [23] found that a delay of 1 or 2 s impaired learning and a delay of 3 s led to no learning at all. Thelen and Smith [30] also stress the value for infant contingency learning of moving, seeing and hearing all being time-locked.

### 2.2 Technical specification

#### 2.2.1 Audio processing (response triggers)

The fundamental frequency range for infants has been identified as between 30 Hz and 2500 Hz [17], however, in order to remove screeches uttered by the infants and to reduce interference from adult voices, the F0 range to elicit responses from the app was set to 250 Hz–750 Hz. The app should not respond to non-voiced sounds which we are not interested in reinforcing (whispers, hissing, and more importantly environmental sounds such as banging, tapping, rustling etc.).

#### 2.2.2 Visual responses

**Fig. 1:**
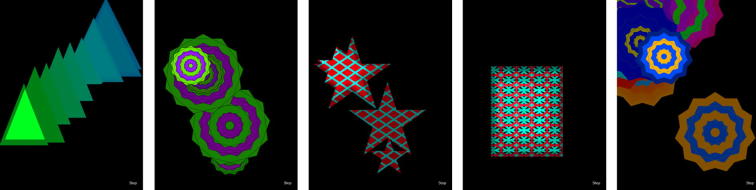
BabbleApp screenshots, demonstrating the variety of shape, size, colour and patterning, as well as patterns of movement across the screen.

Coloured and patterned moving objects with different types of movement were chosen as visual responses to be attractive to the target age group (see Fig. 1).

#### 2.2.3 System efficiency

The app should perform in real-time, or with as short a delay as possible between the infant vocalisation and the visual response. Based on the research on contingency learning discussed in Section 2.1 this must be less than one second.

#### 2.2.4 Default running time

In order to prevent the app from being used as a ‘babysitter’ and replacing real-life interaction between family members, BabblePlay must automatically shut itself off after 5 min.

## 3 Implementation

The audio processing algorithm was originally written in Matlab (2015a) and then implemented as an app which was written in Xcode (10.1) for iOS 8.0.

### 3.1 Audio processing and feature extraction

**Fig. 2:**
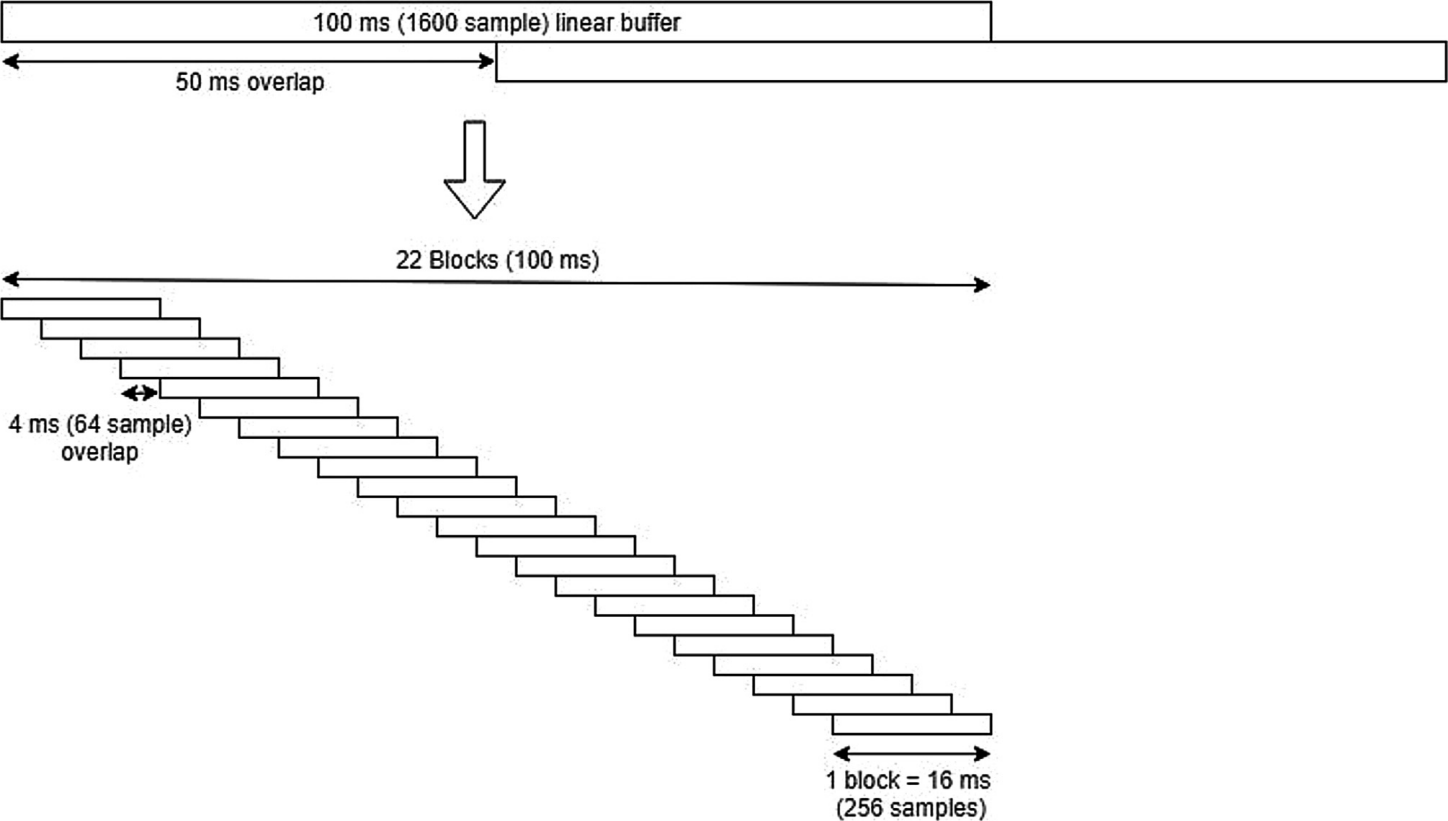
A schematic of the audio processing for BabblePlay.

The acoustic signal is received by the integral microphone of the iPad (Audio in) which is processed at 16 kHz sample rate. A 30 s mono channel circular buffer is populated with a 5 ms input latency. A 100 ms buffer then iterates at 50 Hz (producing a 1600 sample linear buffer, which repopulates every 800 samples after the initial 1600 have been received). This buffer is then divided into 22 blocks of 256 samples with an overlap of 75% which is equal to a hop size of 64 samples/4 ms (see Fig. 2).

Features of fundamental frequency (pitch) and amplitude (volume) are extracted from each block. Pitch is calculated using the Yin algorithm [6], which is based on established autocorrelation methods of pitch extraction for speech signals, modified to not include the parabolic interpolation. Root Mean Square is used to calculate the amplitude of the signal as the characteristic corresponding to the volume of the sound being produced.

**Fig. 3:**
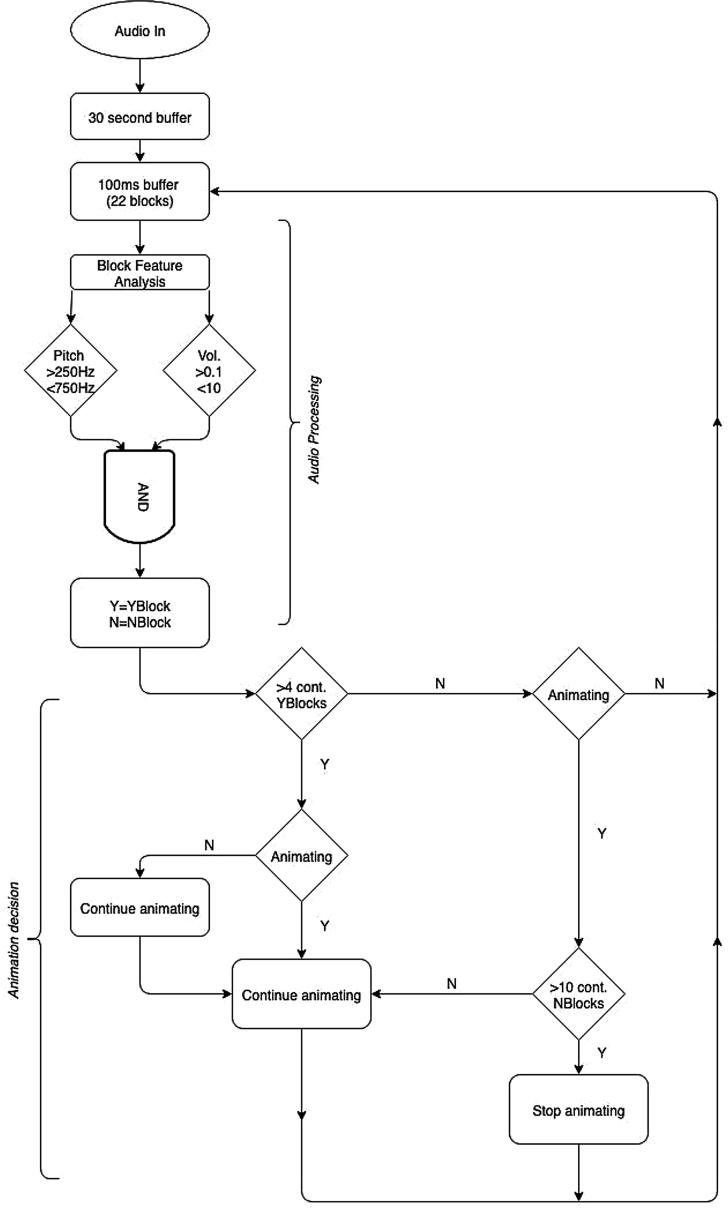
A schematic of BabblePlay.

Fig. 3 shows a schematic for the processing and decision making algorithm implemented in BabblePlay. A binary decision is made for each feature based on parameters of the desired vocalisations: they are considered positive (Y) if the f0 of the given block ≧250 Hz and 750 Hz, and the RMS ≧ 0.1 and < 10. Note that these parameters can be altered in the settings of the app and the volume parameter changed if needed on a case by case basis, depending on distance to the microphone and the specification of the tablet being used. Both features must be positive for that block to be classified as a Yblock, otherwise it is classed as a Nblock. Four consecutive Yblocks (overlapped by 75%) are required for the app to initiate an animation, i.e. for a vocalization to be identified. The animation will continue to appear on the screen until 10 consecutive Nblocks (with an overlap of 75%) are received, at which point the animation disappears and the screen returns to black. New animations are initiated once another four consecutive positive blocks have been received. Once the app has been running for 5 min it automatically closes down.

### 3.2 Animations

Once initialised, animations appear (see Fig. 1), which are randomly generated simple shapes with bright contrasting colours that move around the screen, designed to be attractive to infants under one year of age. The initial size of the shape is randomly chosen. It is then controlled by the RMS value (corresponding to the loudness of the infant’s vocalisation), whereby a larger value increases the size of the shape. We made the size of the shape analogous to the loudness of the vocalisation in order to add another cue, besides duration, for the infants to base their contingency learning on.^1^ Note that BabblePlay does not necessarily reinforce loud utterances over quiet ones, as it merely responds to changes in amplitude by changes in size. The shape generated moves in a random direction around the screen, leaving a ‘trail’ as long as the animation is triggered (see Fig. 1).

### 3.3 Data collection through BabblePlay

In order to facilitate the use of the app for research, a mono audio file (16 kHz sampling frequency) is created of the vocalisations of the infant (all audio captured by the integral microphone of the iPad while the app is on) as well as a count of the number of vocalizations that the app has identified (i.e. the number of animations that were initiated in that session). The .wav file generated at the end of each session is automatically attached to an email which also contains a record of the timestamped animation initiations. This email is sent to the project research account to allow quantitative analysis. The email is only sent once the ‘send’ button is pressed. Families can choose to delete an email if they do not want the sound file to be listened to by the research team. In such a case, the team is notified (in the next email) that a previous recording has taken place, but that the files were not saved. Families who register an infant on the app can opt in to taking part in the research. If they choose to take part, personal data is sent to the research team including the date of birth of the infant and, optionally, postcode and parental education, alongside audio recordings and vocalisation counts from the app. The baby is identified by a code. If families do not opt into the research no files are sent to the research team.

## 4 Validation and feasibility testing with infant users

Initial, informal, piloting with one typically developing six month old infant produced very promising results, with researchers observing that the infant quickly understood the contingency between vocalising and images appearing on the screen (see video: https://www.york.ac.uk/babbleplay/). In order to generalise across the infant population, controlled testing was needed to test the app’s validity, or its ability to identify the target vocalisations it is intended to detect and respond to, and to ensure that infants could learn to control the app.

### 4.1 Feasibility test: methods

A controlled study was conducted to test the app’s validity and its learnability by infants. Sixty infants (mean age 6.5 months) were recorded by BabblePlay in two 5-minute-long trials. In both trials a caregiver and up to two experimenters were with the infants, but the adults avoided interacting with them and did not vocalise. In the solo-play trial the infant played with non-responsive toys and was recorded by BabblePlay in the background, with its screen hidden from the infant. In the experimental trial the infant interacted with BabblePlay (experimental group: n = 30) or with a non-responsive video (control group: n = 30).

### 4.2 Validation – comparing the app’s vocalisation identification to that of humans: findings

**Fig. 4:**
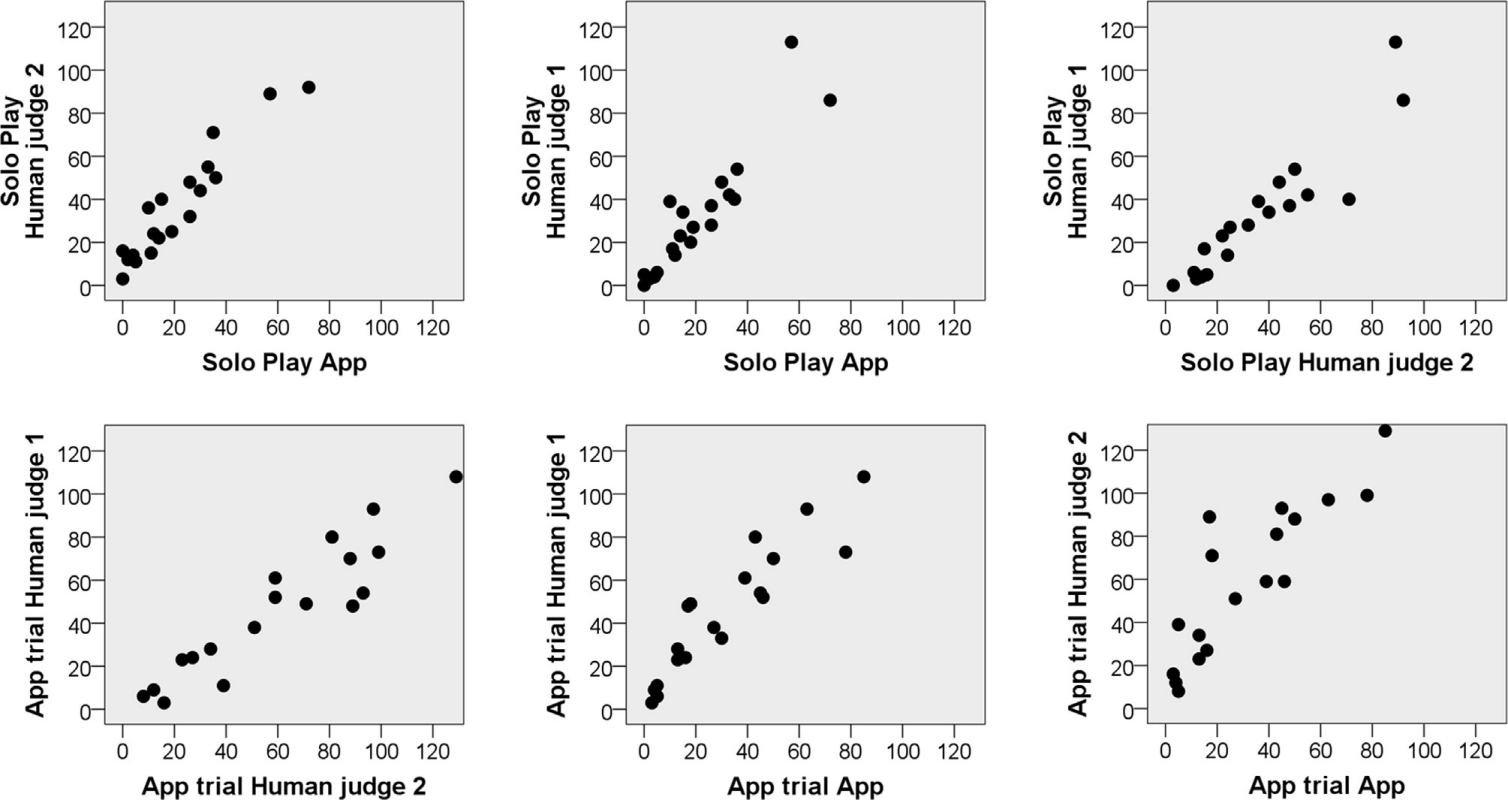
Relations between human and app counts of vocalisations. Top panels: Values for solo-play trials, bottom panels: values for the app trials. As can be seen, the fit between the two humans is very similar to that between each human and the app. The numbers on the axes indicate number of vocalisations identified.

The study produced a dataset for each trial that contained sound files and timestamped counts of the vocalisations which initiated a responses by the app: voiced utterances that do not constitute cries, high pitched squeals or vocal fry. Two human judges were instructed to listen to one third of the sound files, those produced by the last ten infants seen in each group. That amounted to 40 5-minute recording sessions, totalling 200 min of audio. The judges counted infant vocalisations that adhered to the target criteria. Because humans may count a string of syllables as several vocalisations while the app might count them as a single vocalisation, or vice versa, we did not expect the numbers produced by the app and the humans to be the same, but rather, we expected their rankings of some infants as high vocalisers and others as low vocalisers to show positive correlation. The results (see Table 1 and Fig. 4) show that the app and the humans are in close agreement, with the correlations between the two humans (0.93) very similar to those between the humans and the app, in both the solo-play and the app trials: between 0.92 and 0.95 in 3 out of 4 cases, and 0.87 in the fourth. Note, that the vast majority of solo-play trials involved ‘noisy’ toys: Stacking cups, sensory toys which create crinkly or rustling noises, rattles hanging from an arch over a baby seat, etc. App trials, on the other hand, only involved the infant and the iPad. The consistent accuracy of BabblePlay’s counts in both the solo-play trials and the app trials demonstrates that BabblePlay is successfully differentiating between human voices and background noise.

**Table 1:** Table showing the number of vocalisations as counted by humans as compared to those counted by BabblePlay (App).

	Solo Play Trials	App Trials
Correlation Human 1 – Human 2	0.93	0.93
Correlation Human 1 – App	0.92	0.92
Correlation Human 2 – App	0.95	0.87

### 4.3 Feasibility study: Summary of findings

The infants in the experimental group significantly increased their vocalisations throughout the app trial, such that on average only about a third (0.36) of their vocalisations were produced in the first half of the trial and almost two-thirds (0.64) in the second half. In contrast, the control group did not increase their vocalisations in this way, but produced half (0.52) of their vocalisations, on average, in the first half of the trial and half (0.48) in the second. This indicates that infants in the experimental group were indeed learning the contingency between their vocalisations and the animations appearing on the screen, and were motivated to vocalise more as the trial progressed, whereas the control group were not (for a full report of this study see [18], [19]).

## 5 Future work

The current version of the app meets the original specification, and testing has shown that it has potential to be used in a number of situations, including for research. Next steps utilizing the current app include testing the use of the app with clinical populations, to explore the long term impact of BabblePlay on language outcomes in infants who receive the app as a language intervention tool, as well as to test whether infants maintain a high enough level of engagement with the app with repeated use over longer periods.

BabblePlay currently responds to any voiced utterance, not only to utterances which contain consonants (i.e., canonical babble). However, as long as a child has begun to produce canonical babble, then we expect any increase in volubility to be accompanied by an increase in consonant production (if not in relative terms, then in absolute terms). The intention is to further develop the app to include the representation of more features of the vocalisations in the visual feedback (e.g. pitch), and to distinguish between types of vocalisations (e.g. those involving plosives compared to nasals). Our long-term aim is to build an app that responds differentially to different consonant sounds. This presents a significant technological challenge which will be approached by utilising a combination of techniques including implementing Steven’s Landmark’s approach together with neural networks.

## 6 Conclusion

This paper describes the development and validation of an iOS app, BabblePlay, which provides a visual reward for vocal utterances made by an infant in real time, ignoring high pitched screeches, very low-pitched vocalisations and environmental noises. There was good agreement between the number of vocalizations identified by the app and two human judges in 40 BabblePlay recordings. The results of a feasibility study with 60 typically developing infants indicate that the target population can learn the contingency between their vocalisations and images appearing on the screen in a single 5-minute BabblePlay session. This project illustrates the potential for utilising electronic tablet-based games for infants under one year of age, both as a tool for research into infant language development, and for use in the home or in professional environments to demonstrate the impact of immediate feedback for increasing vocalizations in young infants.

## Funding

This work was supported by a C2D2 grant part funded by Wellcome trust, and an EPSRC Impact Accelerator Award.

## Declaration of Competing Interest

The authors declare that they have no known competing financial interests or personal relationships that could have appeared to influence the work reported in this paper.

## Footnotes

1 The app is sensitive to differences in amplitude which the human (adult) ear normalises over, such as the difference in inherent loudness between close vowels and open vowels, or to the silences involved in the closure phase of stop consonants. Therefore, in addition to responding to changes in amplitude that are felt to be analogous to humans’ auditory experience, it also responds to many differences that are not felt as such.
